# Antimicrobial activity of carboxymethyl cellulose–gelatin film containing *Dianthus barbatus* essential oil against aflatoxin‐producing molds

**DOI:** 10.1002/fsn3.1413

**Published:** 2020-01-14

**Authors:** Mehrdad Mohammadi, Mohammad Hossein Azizi, Alaleh Zoghi

**Affiliations:** ^1^ Department of Food Technology Research National Nutrition and Food Technology Research Institute Faculty of Nutrition Sciences and Food Technology Shahid Beheshti University of Medical Sciences Tehran Iran; ^2^ Department of Food Science and Technology Faculty of Agriculture Tarbiat Modarres University Tehran Iran

**Keywords:** Aflatoxin, antimicrobial packaging, CMC, *Dianthus barbatus*, edible film, essential oil

## Abstract

Edible films, as novel degradable materials in food packaging, play an important role in removing consumers' concerns about environmental pollution and food contaminations. Carboxymethyl cellulose (CMC)–gelatin (G) edible films with the ratio 4 to 1 was selected as the optimal film based on physical, mechanical, and physicochemical findings. Then, the effects of 0, 300, 450, and 600 ppm *Dianthus barbatus* essential oil (DbE) on water vapor permeability, tensile strength, elongation at break, water solubility, glass transition temperature, color, oxygen permeability, and antimicrobial activities on the optimal film were investigated. CMC: G (4:1) containing 600 ppm DbE as the antibacterial–antioxidant film was the best formulae (*p* < .05) for preventing three types of aflatoxin‐producing mold including *A. flavus* (PTCC‐5004), *A. parasiticus* (PTCC‐5286), and *A. parasiticus* (PTCC‐5018) on pistachios for 6 months.

## Graphical abstract

Edible films, as novel degradable materials in food packaging, play an important role to removing consumers' concerns about environmental pollution and food contaminations. Carboxymethyl cellulose (CMC)–gelatin (G) edible films with the ratio 4 to 1 containing 600 ppm Dianthus barbatus essential oil as antibacterial‐antioxidant film was the best formulae (*p* < .05) for preventing three types of aflatoxin‐producing mold including A. flavus (PTCC‐5004), A. parasiticus (PTCC‐5286), and A. parasiticus (PTCC‐5018) on pistachios for 6 months. The developed edible film can be used as a biodegradable, low‐cost and high‐performance packaging in the food industry to improve the quality, shelf life, safety, and reduce waste and lost from food.

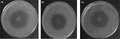

## 1 INTRODUCTION

Edible films are thin layers that are used on the foodstuffs and play a key role in the distribution, marketing, and protection of food products against mechanical damage and microbial and chemical activity. These films are biodegradable, nontoxic, and environmentally friendly (Bourtoom, 2008). Owing to the nonbiodegradable of synthetic polymers, the use of biodegradable materials in the food packaging industry has been considered (Ghanbarzadeh et al., 2007). The use of composite films is one of the most widely used methods to improve the properties of the films produced from one component (Cao, Fu, & He, 2007; Yoo & Krochta, 2011).

Gelatin (G) is obtained from partial destruction of collagen. It has been highly regarded as an edible film, because of its availability, relatively cheap price, biodegradability, and good properties due to its excellent ability to film‐forming and reducing oxygen, oil, and moisture transportation. Also, G has antimicrobial and antioxidant activities (Cao et al., 2007).

Carboxymethyl cellulose (CMC) is used in the packaging industry as a viscosity enhancer and bonding agent. CMC feature for film creation in the food industry is due to its transparency solute, water binding, and viscosity properties (Tongdeesoontorn, Mauer, Wongruong, Sriburi, & Rachtanapun, 2011). Prepared films with this polymer are usually good barrier against gases with intermediate to good mechanical properties but are vulnerable to moisture (Biswal & Singh, 2004; Jahit, Nazmi, Isa, & Sarbon, 2016). Despite the availability and low cost of CMC, the main problem of CMC coatings is its relatively weak inhibition against water vapor (Yoo & Krochta, 2011). The presence of essential oil in formulations of edible films is caused stabilization of phenolic componds and has reduced surface contamination of food through the gradual release of antimicrobial componds (Buonocore et al., 2003). Chouhan, Sharma, and Sanjay Guleria (2017) reported that extract penetrates into lipid membranes and mitochondrial structure of bacteria and mold caused disruption in cellular lipid and subsequently leakage of critical molecules and ions from their cells and impaired electron motor pump.

*Dianthus barbatus* essential oil (DbE) is a pharmaceutical‐decoration plant of the *Carnation* series and *Caryophyllaceae* family which is traditionally used in China, Japan, and Korea as traditional medicine. This plant has some effects such as anti‐inflammatory, anticancer, and antioxidant properties, improving physical, antiviral, antibacterial, antifungal, and anti‐insecticide activities (Chandra & Rawat, 2015; Chandra, Rawat, Chandra, & Rastogi, 2016).

Aflatoxins are natural toxins that are produced by *Aspergillus (A.) flavus* and *A. parasiticus* and are grown in many foodstuffs when their favorable growth conditions are provided (Cheraghali et al., 2007). *A. flavus* (PTCC‐5004) and *A. parasiticus* (PTCC‐5018) produce aflatoxin B_1_, while *A. parasiticus* (PTCC‐5286) produces aflatoxin B_1_, B_2_, G_1_, and G_2_ (Sayanjali, Ghanbarzadeh, & Ghiassifar, 2011). Storage in adverse conditions and moisture absorption from the environment cause mold growth in the nuts (Sabaghi, Maghsoudlou, Khamiri, & Ziaeefar, 2014). Iran is one of the largest manufacturers and exporters of pistachios in the world (Cheraghali et al., 2007). The most important problem of exporting pistachio is increasing aflatoxin during storage due to environmental conditions such as temperature and humidity (Cheraghali et al., 2007; Dini et al., 2013).

Literature searches reveal that no studies documented the effect of CMC‐G containing DbE as an antibacterial–antioxidant compound to the inhibition of producing aflatoxin. Since pistachios are one of the most expensive nuts and consumers prefer the natural antimicrobial agents, the aim of this study was to formulate CMC‐G and DbE edible films using different concentrations of DbE and to determine the antioxidant and antimicrobial properties of these composite on pistachio.

## 2 MATERIALS AND METHODS

### 2.1 Materials

CMC (with an average molecular weight ≅ 50,000) and G (type B, bovine bone, with a bloom value of ~ 300 g) were obtained from Nippon Paper Chemicals Co. (Tokyo, Japan) and Sigma‐Aldrich Co. (St. Louis, USA), respectively. Sunflower oil and DbE were purchased from Behshahr Industrial Co. (Tehran, Iran) and Institute of Medicinal Plants (Karaj, Iran), respectively. Pistachios (*Pistacia vera L*. belongs to the *Anacardiacea* family) and multilayer flexible polystyrene plastics were purchased from a local market (Tehran, Iran). *A. flavus* (PTCC‐5004), *A. parasiticus* (PTCC‐5286), and *A. parasiticus* (PTCC‐5018) were obtained from the culture collection at the Iran Institute of Industrial and Scientific Research. Tween 80, glycerol, physiology serum, and potato dextrose agar (PDA) from Merck Co. (Darmstadt, Germany) were also used.

### 2.2 Methods

#### 2.2.1 CMC‐G film preparation

5% suspension of CMC: G with ratios of 0:5, 1:4, 2.5:2.5, 4:1, and 5:0 were prepared, and after the addition of glycerol 40% (w/w of CMC‐G) as a plasticizer per each gram mixture of CMC‐G and 1% Tween 80 as emulsifier, the mixture was heated for 30 min at 80–90°C, on a hot plate that equipped with a magnetic stirrer with speed of 1,200 rpm, until a clear solution being achieved (F1–F5) (Table 1).

**Table 1:** CMC, gelatin (%), and DbE (ppm) contents used in the formulation of edible films and pistachios uncoated and coated

Formula	CMC (%)	Gelatin (%)	DbE^E^ (ppm)
F1^A^	0	100	–
F2^A^	20	80	–
F3^A^	50	50	–
F4^A^	80	20	–
F5^A^	100	0	–
F6^B^	80	20	–
F7^B^	80	20	300
F8^B^	80	20	450
F9^B^	80	20	600
P1^C^	–	–	–
P2^D^	80	20	300
P3^D^	80	20	450
P4^D^	80	20	600

A Edible films formulated with varying levels of CMC and gelatin: F1–F5.

B Edible films formulated with CMC and gelatin in the ratio 4 to 1 containing different concentrations of DbE: F6–F9.

C Uncoated pistachio as control: P1.

D Coated pistachios with edible films formulated with CMC and gelatin in the ratio 4 to 1 containing different concentrations of DbE: P2, P3, and P4.

E DbE: *Dianthus barbatus* essential oil.

Then, the solution was placed on a glass plate and was heated up to 35°C for 24 hr to be completely dry. Dried films were peeled off the glass petri dishes slowly and maintained in an aluminum foil until usage. Physical, water vapor permeability, and mechanical properties of the developed films were measured to select the optimal film.

#### 2.2.2 Physical properties

##### Transparency

The films were cut into 3 × 2 cm pieces, and then, the pieces of films were placed inside the UV‐Vis Spectrophotometer (Hitachi, Tokyo, Japan). The transparency was determined by measuring the percent light absorption (%T) at 600 nm (Sothornvit, Rhim, & Hong, 2009). The following equation was used to calculate the transparency (T_600_):

(1) $${\text{T}}_{600}=\frac{\text{Log}\;\%\;\text{T}\;\times 100}{d}$$

where d is the film thickness (mm).

##### Thickness

Film thickness was measured in mm with a digital micrometer (Mitutoyo 293–340–30, Tokyo, Japan) with a sensitivity of 0.001 mm to determine the water vapor permeability (WVP) and tensile strength (TS). Results were reported as an average of at least five random locations for each film sheet (Coronado Jorge, Alexandre, Caicedo Flaker, & Bittante, 2015).

#### 2.2.3 Water solubility (WS)

Solubility in water was determined based on the method of Dashipour et al. (2015) with some modifications. The films were cut into 2 × 2 cm; then, the pieces of films were heated in an oven at 100°C for 24 hr to constant weight (Wo). Then, the pieces of films were immersed in containers containing 50 ml of distilled water for 24 hr at room temperature (25°C) on a magnetic stirrer. The films were then filtered by filter paper to obtain undissolved films and dried at 100°C to reach a constant weight to obtain the final dry weight of the film (Wf). The WS was calculated according to the following equation:

(2) $$\text{Water solubility}\;\left(\%\right)=\frac{\text{Wo}-\text{Wf}}{\text{Wo}}\times 100$$

#### 2.2.4 Water vapor permeability (WVP)

WVP was determined using the standard ASTM‐E0096‐005 method. Circular cups with an average diameter of 3 cm and depth of 3.5 cm containing 8 ml of distilled water were sealed by the developed films with rubber gaskets, clips, and grease. Each cup was placed inside a desiccator with silica gel and was weighed every 12 hr (using a digital scale with a precision of 0.0001), and water vapor transport was determined by the weight gain of the cup.

The WVP was calculated according to the following equation:

(3) $$\text{WVP}\;\left({\text{g msPa}}^{-1}\times 10^{-7}\right)=\frac{\Delta m\times x}{A\times \Delta t\times \Delta p}$$

Δm: weight loss per cup (g); X: film thickness (mm); A: area of exposure to the cup (7.06 × 10^−4^ m^2^); Δt: time (s); Δp: the partial pressure difference was 100% (kPa), the RH inside the cup was 100% and the outside of the cup was 0%, which caused a slight partial pressure of 3,179 Pa between the outside and inside of the cup.

#### 2.2.5 Mechanical properties

Mechanical properties of the developed films, including tensile strength also called as tensile stress (TS) expressed in MPa and elongation at break also as called strain (ELB) reported as a percentage relative to comparison flexibility of films (Tabari, 2018), were determined according to Dashipour et al. (2015) with some adjustments using an Instron Universal Testing Machine (model Zwick) according to the ASTM‐D882‐2002. The developed films were cut in 2 × 7 cm pieces and equilibrated at 25°C and 50% RH, and then fixed between the two jaws with an initial distance of 40 mm, and the crosshead speed was set at 10 mm/min and 500 N load cell.

TS was calculated by dividing the peak load in Newton (N) by the cross‐sectional area of the initial film pieces (thickness width in mm^2^).

(4) $$\sigma \left(\text{mP}a\right)=\frac{F}{A}$$

ELB was obtained by the percentage change in length of the film pieces from 40 mm of the original distance between the jaws. ELB is reported as a percentage relative to comparison flexibility of films.

(5) $$\varepsilon \left(\%\right)=\frac{l-{\;l}_{0}}{l_{0}}$$

l: displacement (mm); l_0_: reference length (mm).

#### 2.2.6 Oxygen permeability measurement

Oxygen permeability was measured by the indirect method (Ou, Wang, Tang, Huang, & Jackson, 2005). Fresh and antioxidant‐free sunflower oil was used as a control, and its peroxide value was measured. Wineglasses were filled with sunflower oil, covered with developed film, and stored at 25 ± 2°C and 75% relative humidity for 15 days. The peroxide value (PV) of the pistachios uncoated and coated with edible films formulated containing different concentrations of DbE during 6 months of storage was determined by sodium thiosulfate titration and was calculated using the following equation:

(6) $$\text{Peroxide Value}\;\left({\text{meq O}}^{2}{\;\text{kg}}^{-1}\right)=\frac{V\times N}{M}\times 1000$$

V: consumed thiocyanate volume; N: thiosulfate normality; M: film weight.

#### 2.2.7 Thermal properties

Glass transition temperature (Tg) measurement was performed using Differential Scanning Calorimeter (DSC‐60, Shimadzu, Kyoto, Japan) according to Coronado Jorge et al. (2015) and Bonilla, Bittante, and Sobral (2017) with some adjustments. About 2 mg of developed films were placed in hermetically sealed aluminum pans and heated from − 100 C to + 200°C at a heating rate of 10°C/min, after cooling with liquid nitrogen as a cryogenic. Tg was obtained from the temperature fluctuation point of the temperature.

#### 2.2.8 Color measurement

The color values of developed films in terms of L*, a*, and b* were measured using a colorimeter (Minolta, Tokyo, Japan). Films were placed in desiccators at 25°C and 53% RH prior to optical measurements (Sothornvit et al., 2009). A white reflector standard plate was used as background. Triplicate measurements of color were conducted for each film. Total color difference (ΔE) was calculated by the following equations:

(7) $$\Delta E=\sqrt{{\left({\Delta l}^{*}\right)}^{2}+{\left({\Delta a}^{*}\right)}^{2}+{\left({\Delta b}^{*}\right)}^{2}}$$

ΔL = (L* ‐ L_0_*); Δa = (a* ‐ a_0_*); Δb = (b* ‐ b_0_*); a_0_*, b_0_*, L_0_*: standard color parameter values; a*, b*, L*: film color parameter values; l*: brightness, which has a range of 0 (darkness) to 100 (brightness); a*: from − a*: greenness to + a*: redness; b*: from − b*: blueness to + b* yellowness.

#### 2.2.9 DbE extraction

The hydrodistillation method by the Clevenger apparatus was used to extract DbE. About 200 g *Dianthus barbatus* was ground and then was poured in Clevenger apparatus flasks. After adding distilled water to two‐thirds of flasks, they were attached to the Clevenger device, its contents were heated for 4 hr, and then, essential oil was separated and collected from distilled water.

#### 2.2.10 CMC‐G film containing different DbE concentrations preparation

After CMC‐G film preparation, DbE was added with concentrations of 0, 300, 450, and 600 ppm and stirred for 4 min. Then, the mixture was divided into two sections. One section was used to the immersion of pistachios, and the other one was placed on a plate and was heated up to 35°C for 24 hr to be completely dry. Dried films were peeled off the glass petri dishes slowly and maintained in an aluminum foil until usage.

WVP, mechanical properties, WS, Tg, color, PV, and microbial properties of developed films were measured to select the best antibacterial film for pistachio coating and introduction to the industrial.

#### 2.2.11 Preparation of inocula

Inocula were prepared as described by Sayanjali et al*.* (2011) with some adjustments. The fungal cultures were cultivated on PDA slants for 7 days at 20–25ºC. About 10 ml of this solution was vigorously stirred with crystal balls for several minutes. This allows spores were plucked from colony surface and enter into the solution; then, the solution was passed through No. 1 filter paper in sterile conditions and mold mycelium and plucked particles remained on filter paper. The spores were collected under the filter paper. At this stage, the collected solution was diluted with a solution of Tween 80 (0.1%) and its light absorption was measured using UV‐Vis Spectrophotometer (U‐2000, Hitachi, Japan) at the wavelength of 360 nm to obtain a suspension of 10^4^ spores/ml (Fan & Chen, 1999).

#### 2.2.12 Antimicrobial effects

Antimicrobial activity of developed films was assessed using the agar diffusion method (Dashipour et al., 2015; Sayanjali et al., 2011). The inhibition zone test on PDA was used to determine the antimicrobial activity of developed films containing CMC: G (4:1) with different concentrations of DbE (F6–F9) (Table 1). About 0.1 ml of each mold spore suspension of 10^4^ spores/ml was cultured on PDA. Disk form films of 10 mm diameter were placed in sterile conditions at the center of petri dishes containing a medium. The petri dishes were incubated in the oven at 25°C for 48 hr. Then, the diameter of the inhibitory zone surrounding film disks was measured using a caliper with 0.01 mm precision. Inhibitory zone diameter was considered as an index of antimicrobial activity of films (Sayanjali et al., 2011).

In cases that inhibition zone was not formed means, there was no antimicrobial activity. So, instead of reporting 10 mm (primary diameter of the disk), its equivalent size was considered zero. While the antimicrobial activity was observed, the composed inhibition zone diameter always was more than 10 mm. To ensure the steady growth of mold on the surface of a petri dish, a cultured petri dish without film was used as control. Also, a petri dish lacking mold was used to ensure that no mold contamination there is.

#### 2.2.13 Film application

According to Sayanjali et al. (2011), pistachios were immersed for 2 min in soluble of the films containing CMC: G (4:1) with different concentrations of DbE and then were dried for 24 hr at 35°C before the tests (P2–P4) (Table 1). After drying, coated and uncoated pistachios were stored in polyethylene bags for 6 months at 25°C and then the number of the molds was counted. The average results were expressed in CFU/g.

#### 2.2.14 Statistical analysis

The data were analyzed by one‐way analysis of variance (ANOVA), using SPSS 16 statistical package (SPSS Inc., Chicago, IL, USA). The Duncan test was used to determine statistically significant differences between the means. *p* values < .05 were considered statistically significant for all comparisons. Values were reported as mean ± standard deviation (*SD*) of duplicate treatments (six repetitions for each formula) (Amiri‐Rigi, Mohammadifar, Emam‐Djomeh, & Mohammadi, 2011).

## 3 RESULTS AND DISCUSSION

### 3.1 Physical properties

Table 2 presents the physical properties of CMC‐G film formulae. G significantly decreased transparency and increased WS (*p* < .05). F5 (CMC: G (5:0)) had the highest transparency and lowest WS because of having the lowest level of G compared with the other formulae (*p* < .05), which is due to the nature of the yellowish color of G as described by Ramos et al. (2012).

**Table 2:** Physical and mechanical properties of edible films formulated with varying levels of CMC and gelatin

Formula^A^	Transparency (%)	Thickness (mm)	Water solubility (%)	WVP^B^ (g msPa^−1^ × 10^−7^)	Tensile strength (mPa)	Elongation at break (%)
F1	0.30 ± 0.01^a^	0.100 ± 0.006^a^	51.49 ± 0.21^a^	21.38 ± 0.01^a^	8.34 ± 0.11^a^	31.24 ± 1.34^a^
F2	0.32 ± 0.00^b^	0.101 ± 0.006^a^	36.02 ± 0.43^b^	17.92 ± 0.06^b^	6.14 ± 0.16^b^	35.22 ± 3.37^b^
F3	0.34 ± 0.01^c^	0.094 ± 0.001^a^	31.46 ± 0.74^c^	14.29 ± 0.07^c^	4.62 ± 0.07^c^	41.07 ± 2.34^c^
F4	0.37 ± 0.02^d^	0.100 ± 0.012^a^	28.39 ± 0.29^d^	10.31 ± 0.05^d^	0.75 ± 0.01^d^	55.03 ± 1.46^d^
F5	0.43 ± 0.01^e^	0.097 ± 0.003^a^	21.19 ± 0.85^e^	7.41 ± 0.03^e^	0.23 ± 0.00^e^	60.21 ± 2.54^e^

Note

Means followed by different letters within a column are significantly (*p* < .05) different.

A For formula descriptions see Table 1.

B Water vapor permeability.

The thickness depends on the concentration of the ingredients, the amount of initial solution per unit, the surface, and the rate of pouring on the surface. Since these factors were kept constant, there were no significant differences in thickness (mm) among the formulae containing different levels of CMC and G.

Increasing the amount of G in the formulae led to increase in water solubility due to the instability of the spiral structure and interaction with water, which is in agreement with the findings of Mohanty, Misra, and Hinrichsen (2000).

### 3.2 WVP, TS, and ELB

Films used for food packaging should have low WVP to prevent the exchange of moisture between the food product and the environment. Biodegradable films have poor resistance to moisture due to their hydrophilic properties. WVP decreased when G was combined with the CMC due to their interaction, which leads to an unstable spiral and a more compact structure of the produced film. As shown in Table 2, an increase in the level of CMC significantly yielded a WVP reduction (*p* < .05). F1 (CMC: G (0:5)) had the highest WVP because of the highest level of G compared with the other formulae (*p* < .05), in accordance with the results reported by Sobral and Habitante (2001).

The addition of CMC decreased TS significantly (*p* < .05) and subsequently increased flexibility, because of the formation of strong hydrogen bonds between the CMC chains. The mechanical properties of films depend on the distribution, density of intermolecular, and interactions in the film network. Films of flexibility are required to withstand tensions and strains applied in various applications. Mohanty et al. (2000) found that films produced from CMC are highly flexible and robust.

The interaction between molecular chains of G is high and leads to high TS. The effect of G on TS was also reported by Park (1999), who investigated on methylcellulose and hydroxypropylmethylcellulose films. Mohanty et al. (2000) and Chen and Lai (2008) reported that films developed from CMC had a high TS and G yielded ELB reduction significantly (*p* < .05). Thus, the higher the level of G in the formulae, the lower the flexibility.

### 3.3 Select the optimal film

The optimally developed film was selected using Design‐Expert software version 7 (DX7) (Stat‐Ease Inc., Minneapolis, MN, USA) and a D‐Optimal design. Coefficients between − 2 and + 2 were given to the physical properties, WVP, and mechanical variables based on their importance. Nieto (Nieto, 2009) reported that selecting the best formulae depends on the purposes such as good mechanical strength to prevent tensions during transport, properties of inhibitory to gases and moisture, high flexibility, adaptation to the shape of foodstuff without fracture, and easily used as a coating. The obtained results showed that the film formulated with CMC and G in the ratio 4 to 1 (F4) was the most significantly appropriate (*p* < .05) and was selected as the optimal film. The rest of the experiments carried out on F4 containing different concentrations of DbE (F6‐F9).

### 3.4 WVP, TS, and ELB of F4 containing different concentrations of DbE

According to the results shown in Table 3, the WVP decreased with increasing the concentration of DbE in formulae (*p* < .05). Film‐free DbE (F6) and the film containing 600 ppm of DbE (F9) had the highest and the lowest permeability, respectively (*p* < .05), which is consistent with the findings of Sánchez‐González, Vargas, Gonzalez‐Martinez, Chiralt, and Chafer (2009) and Pérez‐Gago and Rhim (2014). Sánchez‐González, Chiralt, Gonzalez‐Martinez, and Cháfer (2011) reported that adding 3% of bergamot essential oil to chitosan film resulted in a 50% reduction in WVP, due to the increased hydrophobicity of the film containing essential oil.

**Table 3:** Water vapor permeability (WVP), mechanical properties, water solubility, thermal properties, and color values of edible films formulated with CMC and gelatin in the ratio 4 to 1 containing different concentrations of *dianthus barbatus* essential oil

Formula^A^	WVP (g msPa^−1^ × 10^−7^)	Tensile strength (mPa)	Elongation at break (%)	Water solubility (%)	Tg^B^ (^°^C)	b*	a*	L*	ΔE^C^
F6	10.31 ± 0.05^a^	0.75 ± 0.01^a^	55.03 ± 1.46^a^	28.39 ± 0.29^a^	63.34 ± 0.15^a^	3.23 ± 0.94^a^	−1.91 ± 0.28^a^	88.65 ± 0.11^a^	4.17 ± 0.13^a^
F7	7.21 ± 0.02^b^	0.54 ± 0.04^b^	63.52 ± 1.06^b^	21.37 ± 0.94^b^	59.54 ± 0.14^b^	5.19 ± 0.61^b^	−2.34 ± 0.16^b^	87.16 ± 0.25^b^	6.54 ± 0.18^b^
F8	5.48 ± 0.07^c^	0.32 ± 0.01^c^	65.22 ± 1.01^c^	15.36 ± 0.93^c^	58.71 ± 0.01^c^	7.64 ± 0.11^c^	−3.15 ± 0.00^c^	86.43 ± 0.04^c^	8.53 ± 0.04^c^
F9	2.19 ± 0.01^c^	0.16 ± 0.03^d^	68.37 ± 0.01^d^	9.86 ± 0.07^d^	57.69 ± 0.20^d^	8.41 ± 0.16^d^	−3.71 ± 0.01^d^	86.19 ± 0.00^d^	9.72 ± 0.05^d^

Note

Means followed by different letters within a column are significantly (*p* < .05) different.

A For formula descriptions see Table 1.

B Glass transition temperature

C Total color difference

As shown in Table 3, the increase of DbE concentration resulted in a significant decrease in TS and an increase in the ELB (*p* < .05) (Aguirre, Borneo, & Leon, 2013; Sánchez‐González et al., 2009). F6 showed the highest TS and the lowest ELB and F9 showed the lowest TS and the highest ELB, which is in agreement with the findings of Aguirre et al. (2013). The addition of essential oils changed mechanical properties of developed films by the degradation of the film matrix due to changes in the molecular level (Chen & Lai, 2008), their oily nature (Sánchez‐González et al., 2009) or as a polymer emollient (Aguirre et al., 2013). The essential oils weaken the film's inner bonds and increase the flexibility and mobility of polymer chains (Aguirre et al., 2013).

### 3.5 WS and Tg of F4 containing different concentrations of DbE

The increase of DbE concentration resulted in a significant decrease in TS and an increase in the solubility (*p* < .05). According to the results shown in Table 3, F6 showed the highest solubility, and F9 showed the lowest solubility, due to the hydrophobic and nonpolar properties of the DbE, which is in agreement with findings of Maizura, Fazilah, Norziah, and Karim (2007) and Sánchez‐González et al. (2009).

The addition of DbE showed a significant effect on the Tg in all formulae, in accordance with the findings of Bonilla et al. (2017). Tg decreased significantly with the increase of the DbE concentration (*p* < .05) since DbE is as an emollient in the film. The F6 showed the highest Tg and F9 showed the lowest Tg.

### 3.6 Color values of F4 containing different concentrations of DbE

The data presented in Table 3 showed that b* value increased significantly (*p* < .05) with increasing the DbE concentration. So, the F9 and F6 had the highest and lowest b* value, respectively. b* value indicates a yellow‐blue color and a positive b* value indicated that the formulae had a yellow tint. By increasing the concentration of DbE, the brightness and redness of the developed films decreased and their yellowness increased due to the yellow nature of the DbE. Also, a* and L* values of the developed films decreased significantly (*p* < .05).

F6 and F9 had the highest and lowest a* and L* values, respectively. Film color is an important attribute that influences consumer acceptance of the food product and has an important role in its marketability. The increase of ΔE in the formulae containing DbE was associated with an increase in b* and slightly more yellowness than F6. However, the increase in the color values of developed films was not sufficient to reduce their apparent acceptability, in accordance with the findings of Atarés, Pérez‐Masiá, and Chiralt (2011).

### 3.7 PV (meq O2 kg^−1^) of uncoated and coated pistachios with F4 containing different concentrations of DbE during 6 months of storage

PV changing during storage is presented in Table 4. PV in all formulae significantly increased during the storage period (*p* < .05) but with a slight slope in coated pistachios. Different values for the maximum allowable concentration of PV had reported in nuts. UNICEF was reported 5 meq O^2^ kg^−1^ as an acceptable threshold.

**Table 4:** Peroxide value (meq O^2^ kg^−1^) of pistachios uncoated and coated with edible films formulated with CMC and gelatin in the ratio 4 to 1 containing different concentrations of *dianthus barbatus* essential oil during 6 months of storage

Formula^A^	Storage time (months)					
	1	2	3	4	5	6
P1	0.5 ± 0.01^a^	1.1 ± 0.03^a^	1.6 ± 0.06^a^	2 ± 0.02^a^	3.4 ± 0.05^a^	6.7 ± 0.04^a^
P2	0.4 ± 0.02^b^	0.9 ± 0.02^b^	1.1 ± 0.01^b^	1.3 ± 0.04^b^	2.9 ± 0.01^b^	3.5 ± 0.02^b^
P3	0.3 ± 0.02^c^	0.7 ± 0.01^c^	0.9 ± 0.01^c^	1.1 ± 0.01^c^	2.5 ± 0.02^c^	3.1 ± 0.01^c^
P4	0.1 ± 0.01^d^	0.6 ± 0.04^d^	0.8 ± 0.02^d^	1 ± 0.03^d^	1.3 ± 0.05^d^	1.9 ± 0.02^d^

Note

Values followed by different letters within a column are significantly (*p* < .05) different.

A For formula descriptions see Table 1.

The amount of oxidation during 6 months was lower in coated pistachios, which means that the developed films could reduce the oxidation of pistachios to lower than the allowed threshold (*p* < .05). Oxidation rate in coated pistachios was lower during the time which means that developed films were able to reduce the oxidation rate of pistachio. Increasing the concentration of DbE in developed films yielded a slight slope in pistachio oxidation during storage. Bonilla, Atares, Vargas, and Chiralt (2012)) reported that G edible coating effectively influenced the oxidation rate, due to the good inhibitory effect of protein coatings such as G against oxygen penetration. The findings of Vanaei, Sedaghat, Abbaspour, Kaviani, and Azarbad (2014) showed that the use of aloe vera alone is not a good coating for pistachio conservation, but the film containing chitosan 0.5% is more suitable for pistachio conservation against oxygen penetration.

### 3.8 Antimicrobial properties of F4 containing different concentrations of DbE on PDA

The inhibitory activity of developed films was measured based on measurements of composed halo diameter around the disk‐shaped film. As shown in Table 5, the diameter of the inhibition zone of F6 was zero which showed no antimicrobial activity. According to the diameter of the inhibitory zone, the highest and the lowest antimicrobial activity of the DbE were against *A. parasiticus* (PTCC‐5286) and *A. flavus* (PTCC‐5004), respectively.

**Table 5:** Antimicrobial activity of edible films formulated with CMC and gelatin in the ratio 4 to 1 containing different concentrations of *dianthus barbatus* essential oil on PDA

Formula^A^	Inhibitory zone diameter (mm)		
	*A. flavus* (PTCC−5004)	*A. parasiticus* (PTCC−5286)	*A. parasiticus* (PTCC−5018)
F6	0^B^	0	0
F7	3.19 ± 0.01^a^	5.21 ± 0.03^a^	3.32 ± 0.02^a^
F8	4.35 ± 0.02^b^	7.31 ± 0.08^b^	5.25 ± 0.04^b^
F9	6.87 ± 0.05^c^	10.91 ± 0.03^c^	7.34 ± 0.06^c^

Note

Values followed by different letters within a column are significantly (*p* < .05) different.

A For formula descriptions see Table 1.

B No inhibitory zone surrounding film disks formed represents the CMC‐G film without a DbE has no antimicrobial properties.

**Figure 1:**
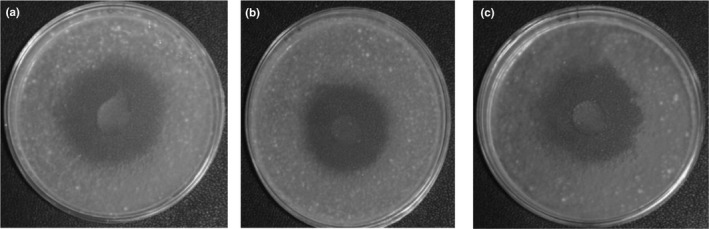
Antimicrobial activities against (a) *A. flavus* (PTCC‐5004); (b) *A. parasiticus* (PTCC‐5286); and (c) *A. parasiticus* (PTCC‐5018) of edible films formulated with CMC and gelatin in the ratio 4 to 1 containing 600 ppm of *dianthus barbatus* essential oil on PDA

The zone of inhibition was increased significantly by the addition of DbE at concentrations of 300, 450, and 600 ppm (*p* < .05). This indicates that the films containing DbE (F7‐F9) were effective against *A. flavus* (PTCC‐5004), *A. parasiticus* (PTCC‐5286), and *A. parasiticus* (PTCC‐5018). Antimicrobial activity of F9 formulae against three mentioned types of mold is shown in Figure 1. This is consistent with the Begum, Mahmud, Naimul Islam, and Khan (2018) findings that report essential oils, as antimicrobial and antioxidant compounds and natural additives, can be applied to packaging materials to inhibit microbial contamination and thus increase the shelf life of the product.

### 3.9 Antimicrobial effects of F4 containing different concentrations of DbE on pistachio after 6 months of storage

**Figure 2:**
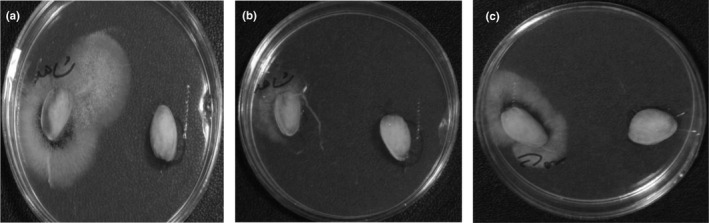
Aflatoxin‐producing by a) *A. flavus* (PTCC‐5004); b) *A. parasiticus* (PTCC‐5286); and c) *A. parasiticus* (PTCC‐5018) on uncoated (left) and coated (right) pistachios with edible films formulated with CMC and gelatin in the ratio 4 to 1 containing 600 ppm of *dianthus barbatus* essential oil

According to the results shown in Table 6, coated pistachios showed no growth of aflatoxin‐producing mold in 3 concentrations of 300, 450, and 600 ppm of DbE (P2‐P4), while the uncoated pistachio (P1) showed a mild growth. Aflatoxin‐producing by *A. flavus* (PTCC‐5004), *A. parasiticus* (PTCC‐5286), and *A. parasiticus* (PTCC‐5018) on uncoated and coated pistachios with F9‐formulated edible films is shown in Figure 2. Thus, antimicrobial coating containing each of the three concentrations of DbE well prevented the growth of *A. flavus* (PTCC‐5004), *A. parasiticus* (PTCC‐5286), and *A. parasiticus* (PTCC‐5018) after 6 months in mold environment, which is in accordance with Galus and Kadzinska (2015) and Begum et al. (2018) findings.

**Table 6:** Aflatoxin‐producing mold count of uncoated and coated pistachios with edible films formulated with CMC and gelatin in the ratio 4 to 1 containing different concentrations of *dianthus barbatus* essential oil after 6 months of storage in polyethylene bags

Formula^A^	Mold count (CFU/g)		
	*A. flavus* (PTCC−5004)	*A. parasiticus* (PTCC−5286)	*A. parasiticus* (PTCC−5018)
P1	4.1 × 10^5^	1.6 × 10^6^	3.2 × 10^5^
P2	NDB	ND	ND
P3	ND	ND	ND
P4	ND	ND	ND

A For formula descriptions see Table 1.

B ND: Not detected.

## 4 CONCLUSIONS

The results showed that changes in CMC‐G ratios in formulated edible films had a significant effect on its mechanical and physical–chemical properties. As a result of increasing the amount of CMC, the film's characteristics improved. Among the developed formulae, the optimal film was the film formulated with CMC and G in the ratio 4 to 1 (F4). In order to evaluate the mechanical, physicochemical, antioxidant, and antimicrobial properties of the optimal film, 300, 450, and 600 ppm of DbE was added.

The addition of 3 mentioned concentrations of DbE to the optimal film could show an antimicrobial effect against *A. flavus* (PTCC‐5004), *A. parasiticus* (PTCC‐5286), and *A. parasiticus* (PTCC‐5018) at the end of 6 months of storage. In terms of antioxidant and antimicrobial properties, the most appropriate and most affordable concentration that has the more inhibitor effect of DbE was 600 ppm. So, F9 was selected as the best formulae for pistachio protection against aflatoxin‐producing molds. Thus, F9 can be used as a biodegradable, low‐cost, and high‐performance packaging in the food industry to improve the quality, shelf life, safety, and reduce waste and loss of food.

## CONFLICT OF INTEREST

The authors declare no conflicts of interest.

## ETHICAL APPROVAL

We declare no ethical issue related with this article.

## References

[fsn31413-bib-0001] Aguirre, A. , Borneo, R. , & Leon, A. E. (2013). Antimicrobial, mechanical and barrier properties of triticale protein films incorporated with oregano essential oil. Food Bioscience, 1, 2–9. 10.1016/j.fbio.2012.12.001

[fsn31413-bib-0002] Amiri‐Rigi, A. , Mohammadifar, M. A. , Emam‐Djomeh, Z. , & Mohammadi, M. (2011). Response surface optimisation of spray dryer operational parameters for low‐phenylalanine skim milk powder. International Journal of Food Science & Technology, 46(9), 1830–1839. 10.1111/j.1365-2621.2011.02688.x.

[fsn31413-bib-0004] Atarés, L. , Pérez‐Masiá, R. , & Chiralt, A. (2011). The role of some antioxidants in the HPMC film properties and lipid protection in coated toasted almonds. Journal of Food Engineering, 104(4), 649–656. 10.1016/j.jfoodeng.2011.02.005

[fsn31413-bib-0005] Begum, T. , Mahmud, J. , Naimul Islam, M. D. , & Khan, R. A. (2018). Essential oils and biodegradable packaging materials: Application on food preservations. Scientific Review, 5(1), 1–7.

[fsn31413-bib-0006] Biswal, D. R. , & Singh, R. P. (2004). Characterization of carboxymethyl cellulose and polyacrylamide graft copolymer. Carbohydrate Polymers, 57(4), 379–387. 10.1016/j.carbpol.2004.04.020

[fsn31413-bib-0007] Bonilla, J. , Atares, L. , Vargas, M. , & Chiralt, A. (2012). Edible films and coatings to prevent the detrimental effect of oxygen on food quality: Possibilities and limitations. Journal of Food Engineering, 110(2), 208–213. 10.1016/j.jfoodeng.2011.05.034

[fsn31413-bib-0008] Bonilla, J. , Bittante, A. M. Q. B. , & Sobral, P. J. A. (2017). Thermal analysis of gelatin–chitosan edible film mixed with plant ethanolic extracts. Journal of Thermal Analysis and Calorimetry, 130(2), 1221–1227. 10.1007/s10973-017-6472-4

[fsn31413-bib-0009] Bourtoom, T. (2008). Edible films and coatings: Characteristics and properties. International Food Research Journal, 15(3), 237–248.

[fsn31413-bib-0010] Buonocore, C. G. , Nobile, M. A. , Panizza, A. , Bove, S. , Batatglia, G. , & Nicolais, L. (2003). Modeling the lysozyme release kinetics from antimicrobial films intended for food packaging applications. Journal of Food Science, 68(4), 1365–1370. 10.1111/j.1365-2621.2003.tb09651.x.

[fsn31413-bib-0011] Cao, N. , Fu, Y. , & He, J. (2007). Mechanical properties of gelatin films cross‐linked, respectively, by ferulic acid and tannin acid. Food Hydrocolloids, 21(4), 575–584. 10.1016/j.foodhyd.2006.07.001.

[fsn31413-bib-0012] Chandra, S. , & Rawat, D. S. (2015). Medicinal plants of the family Caryophyllaceae: A review of ethno‐medicinal uses and pharmacological properties. Integrative Medicine Research, 4(3), 123–131. 10.1016/j.imr.2015.06.004. 28664118 PMC5481791

[fsn31413-bib-0013] Chandra, S. , Rawat, D. S. , Chandra, D. , & Rastogi, J. (2016). Nativity, phytochemistry, ethnobotany and pharmacology of dianthus caryophyllus. Research Journal of Medicinal Plant, 10(1), 1–9. 10.3923/rjmp.2016.1.9.

[fsn31413-bib-0014] Chen, C.‐H. , & Lai, L.‐S. (2008). Mechanical and water vapor barrier properties of tapioca starch/decolorized hsian‐tsao leaf gum films in the presence of plasticizer. Food Hydrocolloids, 22(8), 1584–1595. 10.1016/j.foodhyd.2007.11.006.

[fsn31413-bib-0015] Cheraghali, A. M. , Yazdanpanah, H. , Doraki, N. , Abouhossain, G. , Hassibi, M. , Ali‐abadi, S. , … Zamanian, F. (2007). Incidence of aflatoxins in Iran pistachio nuts. Food and Chemical Toxicology, 45(5), 812–816. 10.1016/j.fct.2006.10.026. 17161513

[fsn31413-bib-0016] Chouhan, S. , Sharma, K. , & Sanjay Guleria, S. (2017). Antimicrobial activity of some essential oils‐present status and future perspectives. Medicines, 4, 1–21. 10.3390/medicines4030058 PMC5622393 28930272

[fsn31413-bib-0017] Coronado Jorge, M. F. , Alexandre, E. M. C. , Caicedo Flaker, C. H. , Bittante, A. M. Q. B. , & Sobral, P. J. d. A. (2015). Biodegradable films based on gelatin and montmorillonite produced by spreading. International Journal of Polymer Science, 3, 1–9. 10.1155/2015/806791

[fsn31413-bib-0018] Dashipour, A. , Razavilar, V. , Hosseini, H. , Shojaee‐Aliabadi, S. , Germand, B. , Ghanati, K. , … Khaksar, R. (2015). Antioxidant and antimicrobial carboxymethyl cellulose films containing Zataria multiflora essential oil. International Journal of Biological Macromolecules, 72, 606–613. 10.1016/j.ijbiomac.2014.09.006. 25220790

[fsn31413-bib-0019] Dini, A. , Khazaeli, P. , Roohbakhsh, A. , Madadlou, A. , Pourenamdari, M. , Setoodeh, L. , … Khodadadi, E. (2013). Aflatoxin contamination level in Iran’s pistachio nut during years 2009–2011. Food Control, 30, 540–544. 10.1016/j.foodcont.2012.08.012.

[fsn31413-bib-0020] Fan, J. J. , & Chen, J. H. (1999). Inhibition of Aflatoxin‐producing fungi by Welsh onion extracts. Journal of Food Protection, 62(4), 414–417. 10.4315/0362-028X-62.4.414. 10419218

[fsn31413-bib-0021] Galus, S. , & Kadzinska, J. (2015). Food applications of emulsion‐based edible films and coatings. Trends in Food Science and Technology, 45(2), 273–283. 10.1016/j.tifs.2015.07.011.

[fsn31413-bib-0022] Ghanbarzadeh, B. , Musavi, M. , Oromiehie, A. R. , Rezayi, K. , Razmi Rad, E. , & Milani, J. (2007). Effect of plasticizing sugars on water vapor permeability, surface energy and microstructure properties of zein films. LWT‐ Food Science and Technology, 40(7), 1191–1197. 10.1016/j.lwt.2006.07.008.

[fsn31413-bib-0023] Jahit, I. S. , Nazmi, N. N. M. , Isa, M. I. N. , & Sarbon, N. M. (2016). Preparation and physical properties of gelatin/CMC/chitosan composite films as affected by drying temperature. International Food Research Journal, 23(3), 1068–1074.

[fsn31413-bib-0024] Maizura, M. , Fazilah, A. , Norziah, M. H. , & Karim, A. A. (2007). Antibacterial activity and mechanical of partially hydrolyzed properties of sago starch‐alginate edible film containing lemon grass essential oil. Journal of Food Science, 72(6), 324–330. 10.1111/j.1750-3841.2007.00427.x 17995673

[fsn31413-bib-0025] Mohanty, A. K. , Misra, M. , & Hinrichsen, G. (2000). Biofibres, biodegradable polymers and biocomposites: An overview. Macromolecular Materials and Engineering, 276(277), 1–24. 10.1002/(SICI)1439-2054(20000301)276:1<1:AID-MAME1>3.0.CO;2-W.

[fsn31413-bib-0026] Nieto, B. N. (2009). Structure and Function of Polysaccharide Gum‐Based Edible Films and Coatings In Embuscado M., & Huber K. C. (Eds.), Edible Films and Coatings for Food Applications (pp. 57–113). New York, USA: Springer.

[fsn31413-bib-0027] Ou, S. , Wang, Y. , Tang, S. , Huang, C. , & Jackson, M. G. (2005). Role of ferulic acid in preparing edible films from soy protein isolate. Journal of Food Engineering, 70(2), 205–210. 10.1016/j.jfoodeng.2004.09.025.

[fsn31413-bib-0028] Park, H. J. (1999). Development of advanced edible coatings for fruits. Trends in Food Science and Technology, 10(8), 254–260. 10.1016/S0924-2244(00)00003-0

[fsn31413-bib-0029] Pérez‐Gago, M. B. , & Rhim, J. W. (2014). Edible coating and film materials: Lipid bilayers and lipid emulsions In Innovations in Food Packaging; 2nd ed.; Han J. H. Ed.; Academic Press, Elsevier, London, Uk, pp. 325–350.

[fsn31413-bib-0030] Ramos, O. L. , Pereira, J. O. , Silva, S. I. , Fernandes, J. C. , Franco, M. I. , Lopes‐da‐Silva, J. A. , … Malcata, F. X. (2012). Evaluation of antimicrobial edible coatings from a whey protein isolate base to improve the shelf life of cheese. Journal of Dairy Science, 95(11), 6282–6292. 10.3168/jds.2012-5478 22939797

[fsn31413-bib-0031] Sabaghi, M. , Maghsoudlou, A. , Khamiri, M. , & Ziaeefar, A. (2014). The effect of coating of chitosan incorporating and green tea extract on shelf life of walnut kernel. Journal of Research and Innovation in Food Science and Technology, 3(4), 361–374.

[fsn31413-bib-0032] Sánchez‐González, L. , Chiralt, A. , Gonzalez‐Martinez, C. , & Cháfer, M. (2011). Effect of essential oils on properties of film forming emulsions and films based on hydroxypropylmethylcellulose and chitosan. Journal of Food Engineering, 105(2), 246–253. 10.1016/j.jfoodeng.2011.02.028

[fsn31413-bib-0033] Sánchez‐González, L. , Vargas, M. , Gonzalez‐Martinez, C. , Chiralt, A. , & Chafer, M. (2009). Characterization of edible films based on hydroxyl propyl methyl cellulose and tea tree essential oil. Food Hydrocolloids, 23, 2102–2109. 10.1016/j.foodhyd.2009.05.006

[fsn31413-bib-0034] Sayanjali, S. , Ghanbarzadeh, B. , & Ghiassifar, S. H. (2011). Evaluation of antimicrobial and physical properties of edible film based on carboxymethyl cellulose containing potassium sorbate on some mycotoxigenic Aspergillus species in fresh pistachios. LWT ‐ Food Science and Technology, 44, 1133–1138. 10.1016/j.lwt.2010.12.017

[fsn31413-bib-0035] Sobral, P. J. A. , & Habitante, A. M. Q. B. (2001). Phase transitions of pigskin gelatin. Food Hydrocolloids, 15(4), 377–382. 10.1016/S0268-005X(01)00060-1

[fsn31413-bib-0036] Sothornvit, R. , Rhim, J.‐W. , & Hong, S.‐I. (2009). Effect of nano‐clay type on the physical and antimicrobial properties of whey protein isolate/clay composite films. Journal of Food Engineering, 91, 468–473. 10.1016/j.jfoodeng.2008.09.026

[fsn31413-bib-0037] Tabari, M. (2018). Characterization of a new biodegradable edible film based on Sago Starch loaded with Carboxymethyl Cellulose nanoparticles. Nanomedicine Research Journal, 3(1), 25–30.

[fsn31413-bib-0038] Tongdeesoontorn, W. , Mauer, L. J. , Wongruong, S. , Sriburi, P. , & Rachtanapun, P. (2011). Effect of carboxymethyl cellulose concentration on physical properties of biodegradable cassava starch‐based films. Chemistry Central Journal, 5(1), 1–8. 10.1186/1752-153X-5-6 21306655 PMC3041729

[fsn31413-bib-0039] Vanaei, M. , Sedaghat, N. , Abbaspour, H. , Kaviani, M. , & Azarbad, H. R. (2014). Novel edible coating based on Aloe Vera gel to Maintain pistachio Quality. International Journal of Scientific Engineering and Technology, 3(8), 1016–1019.

[fsn31413-bib-0040] Yoo, S. , & Krochta, J. M. (2011). Whey protein– polysaccharide blended edible film formation and barrier, tensile, thermal and transparency properties. Journal of the Science of Food and Agriculture, 91(14), 2628–2636. 10.1002/jsfa.4502 21717463

